# High-Fat Fish Oil Diet Prevents Hypothalamic Inflammatory Profile in Rats

**DOI:** 10.1155/2013/419823

**Published:** 2013-02-28

**Authors:** Gustavo Duarte Pimentel, Fábio Santos Lira, José César Rosa, Cláudia Maria Oller do Nascimento, Lila Missae Oyama, Regina Lúcia Harumi Watanabe, Eliane Beraldi Ribeiro

**Affiliations:** Departamento de Fisiologia, Disciplina de Fisiologia da Nutrição, Universidade Federal de São Paulo (UNIFESP), 04023-062 São Paulo, SP, Brazil

## Abstract

Whether PUFA diets affect inflammatory mediators in central and peripheral sites is not clear. We investigated the effect of high-fat PUFA diets on the expression of proteins involved in inflammatory pathways in hypothalamus, muscle, and liver. Male rats were fed for 2 months with either chow or high-fat diets enriched with either soy (n-6 PUFAs) or fish oil (n-3 PUFAs). The fish group had normal body weight, low serum NEFA, reduced hypothalamic levels of TNF-**α**, IL-6, and TRAF6, and increased levels of IL-10 receptor. In contrast, the soy group had increased body weight and hypothalamic levels of TRAF6 and NF**κ**Bp65. In muscle, the fish diet reduced TNF-**α** and IL-6 levels. Both PUFA diets increased muscle IL-10 levels and reduced liver TNF-**α** and IL-6 levels. The data showed that the high-fat soy diet induced activation of the hypothalamic NF**κ**B inflammatory pathway, a feature predisposing to feeding and energy expenditure disturbances associated with the development of obesity. On the other hand, the high-fat fish diet improved the central and the peripheral inflammatory profile via reduction of intracellular inflammatory mediators, suggesting a protection against obesity.

## 1. Introduction

Obesity is known to present an inflammatory process of low grade, with elevated levels of cytokines such as interleukin 6 (IL-6), tumor necrosis factor alpha (TNF-*α*), and interleukin 1 beta (IL-1*β*), contributing to the pathogenesis of important disturbances of the obese condition, as insulin resistance and metabolic defects. This inflammatory state has been described to induce elevated signaling through the toll-like receptors TLR2 and TLR4, with activation of the nuclear factor *κ*B (NF*κ*B) pathway in muscle, liver, and adipose tissue [1–4].

In this inflammatory pathway, TLR2/4 binding to the myeloid differentiation factor-88 (MyD88) leads, after some intermediate steps, to the recruitment of the tumor necrosis factor receptor-associated factor-6 (TRAF6). Its interactions with several proteins leads to phosphorylation of the inhibitory factor I*κ*B, which is then targeted for proteosomal degradation, releasing NF*κ*B, whose p65 subunit undergoes phosphorylation and translocates to the nucleus, where it binds to its target genes to produce proinflammatory cytokines [5].

Importantly, prolonged intake of saturated or trans fats has also been associated with NF*κ*B/MyD88 pathway-mediated induction of inflammatory cytokines in the hypothalamus and cytokine-induced impairment of central insulin hypophagia [6–10]. The hypothalamus is a key regulator of energy homeostasis, through the production of orexigenic and anorexigenic neuropeptides targeted by the peripheral hormones leptin and insulin, which exert a pivotal control food intake and energy expenditure. We have shown that the disruption of these hypothalamic mechanisms, including insulin, leptin, and serotonin systems, is associated with obesity [11–16].

The consequences of the dietary consumption of excess polyunsaturated fats to the central control of energy homeostasis have been addressed by few studies. We have recently explored this matter and found that a high-fat diet containing soy oil, a source of omega-6 polyunsaturated fatty acids (PUFAs), induced an obesogenic profile of hypothalamic neuronal activation, as evaluated by c-Fos immunoreactivity [16]. On the other hand, a fish oil-enriched diet, a source of omega-3 PUFAs, abolished serotonin-induced hypophagia and impaired hypothalamic serotonin turnover and 5-HT 2C receptor levels [17]. We have also shown that the high-fat soy-oil diet inhibited insulin-induced hypophagia and Akt serine phosphorylation, while the fish-oil diet prevented these alterations [18]. Whether these divergent effects of the diets are linked to differential inflammatory signaling in the hypothalamus has not been explored.

The aim of the present study was to investigate in rats the effects of the long-term consumption of high-fat diets, enriched with either soy oil (n-6 PUFAs) or fish oil (n-3 PUFAs), on the hypothalamic expression of proteins involved in the inflammatory pathway. 

Additionally, liver and muscle tissue were studied, since little is known about the effects of polyunsaturated fat diets on the modulation of inflammatory status in these tissues. 

## 2. Materials and Methods

### 2.1. Animals and Diets

The Experimental Research Committee of the Federal University of São Paulo approved all animal procedures. They were housed five per cage under 12 h light-dark cycle, at 22 ± 1°C, and with free access to food and water.

The 2-month-old male Wistar rats were randomly assigned to receive one of three *ad libitum *diet treatments for 8 weeks: balanced chow (2.8 kcal/g, 15% kcal from fat, Nuvilab, Brazil) or high-fat diets (3.5 kcal/g, 50% kcal from fat) enriched with 20% (w/w) of either soy oil (Liza, Cargill Agrícola, Brazil) or fish oil (ROPUFA “75” *ω*-3, Roche, DSM Nutritional Products, Brazil) to the standard chow. Casein was added to both high-fat diets (20%, w/w), to achieve the same protein content of the control chow, and butylated hydroxytoluene (0.02% of added oil) was added as an antioxidant agent. The fatty acid composition of the diets was determined by gas chromatography, as previously detailed [12, 18]. The data showed a high percentage of n-3 PUFAs in the fish diet, mainly eicosapentaenoic acid (EPA) and docosahexaenoic acid (DHA), whereas the soy and the control diets had high percentages of n-6 PUFAS, mainly linoleic acid (LA). At the end of the 8-week diet treatment, the rats were overnight fasted and decapitated. Blood and tissues were collected and processed as explained here in after.

### 2.2. Serum Nonesterified Fatty Acids and Endotoxin Levels

Serum nonesterified free fatty acids (NEFA) levels were quantified by colorimetric method (ZenBio, NC, USA). Serum endotoxin was assayed using chromogenic limulus amebocyte lysate kit (Cambrex Bio Science, MD, USA), as previously detailed [9].

### 2.3. Determination of Tissue Cytokines Levels by ELISA

The hypothalamus, liver, and gastrocnemius muscle were homogenized and centrifuged at 12, 000 g for 40 min at 4°C. Protein concentration of the supernatants was determined using BCA assay (Bio-Rad, Hercules, California, USA) with bovine serum albumin as standard. TNF-*α*, IL-1*β*, IL-6, and IL-10 contents were assayed by ELISA in 100 *μ*L aliquots (DuoSet ELISA, R&D Systems, Minneapolis, MN, USA), following the recommendations of the manufacturer. All samples were run in duplicate.

### 2.4. Determinations of Hypothalamic Protein Levels by Western Blotting

The hypothalamus was homogenized in solubilization buffer containing protease inhibitors and centrifuged at 12,000 g for 35 min at 4°C. The protein concentration of the supernatants was determined by BCA assay. 75 *μ*g of protein was resolved in 8 or 10% SDS-PAGE and electrotransferred to nitrocellulose membranes. The membranes were incubated overnight at 4°C with primary antibody against TNF*α*-R, IL-6R, IL-10R, TLR2, TLR4, MyD88, TRAF6, NF*κ*Bp50, or NF*κ*Bp65 (Santa Cruz Biotechnology, CA, USA). The blots were subsequently incubated with peroxidase-conjugated secondary antibody. The bands detected by chemiluminescence were quantified by optical densitometry of developed autoradiographs (Scion Image software, Scion Corporation, Frederick, USA). For the evaluation of protein loading, all membranes were stripped and reblotted with anti-alpha-tubulin primary antibody.

### 2.5. Statistical Analysis

The statistical analysis was performed using the GraphPad Prism statistics software package version 5.0 for Windows (GraphPad Software, San Diego, CA, USA). The data are expressed as means ± SEM. Comparisons among control, soy, and fish groups were performed by one-way ANOVA followed by Tukey's test for multiple comparisons. A value of *P* < 0.05 was considered statistically significant.

## 3. Results

### 3.1. Body Mass and Serum Levels of Nonesterified Fatty Acids and Endotoxin

**Figure 1:**
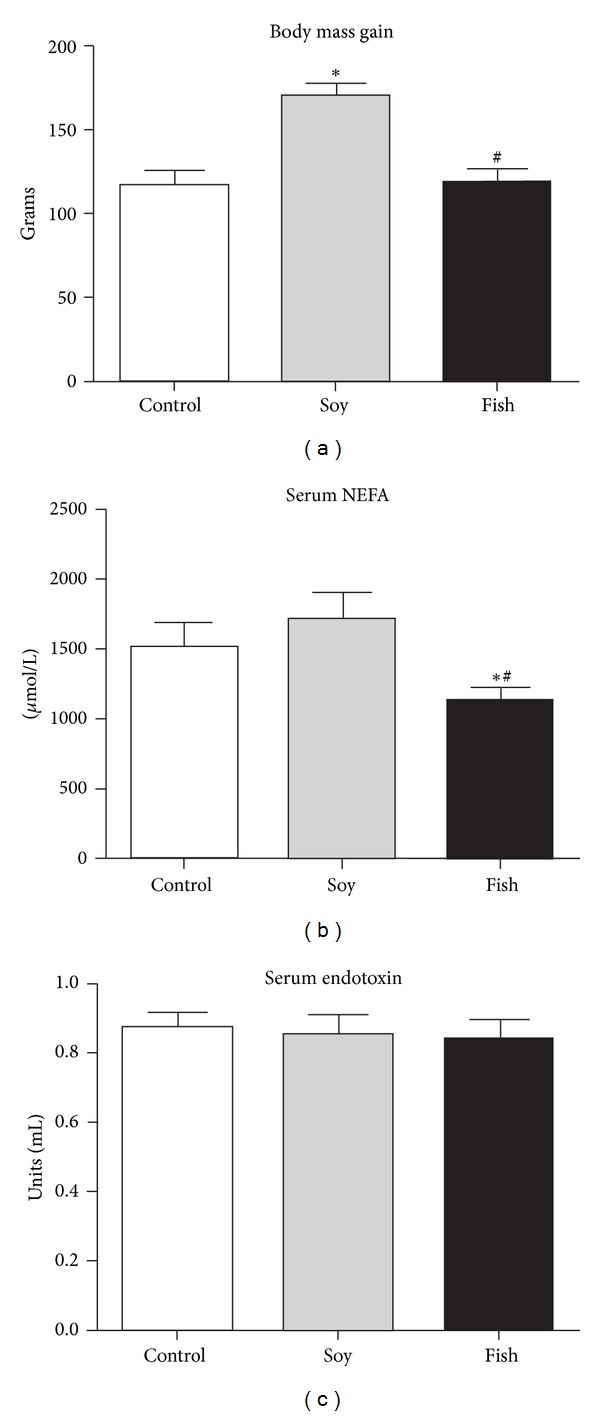
Body mass gain (a) and serum levels of nonesterified fatty acids (b) and endotoxin (c) of rats fed with control (*n* = 6), soy (*n* = 5), or fish diet (*n* = 8). *P < 0.05 versus control; ^#^ *P* < 0.05 soy versus fish.

At the end of diet treatment, body mass was significantly higher in the soy group than that in both the control (+45%) and the fish oil (+42%) groups (Figure 1(a)).

The fish oil group had significantly lower serum NEFA levels than both the control (−22%) and the soy oil (−39%) groups (Figure 1(b)). Serum endotoxin levels were similar among the groups (Figure 1(c)).

### 3.2. Inflammatory Signaling in the Hypothalamus

**Figure 2:**
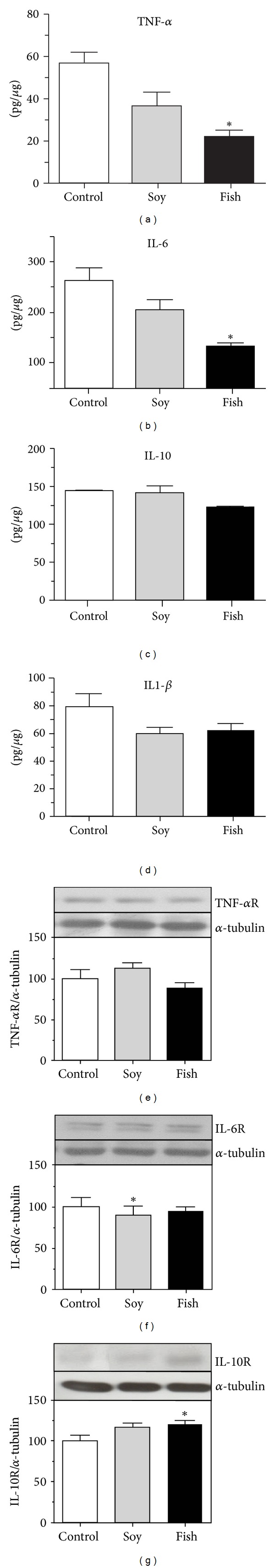
Hypothalamic protein levels of TNF-*α* (a), IL-6 (b), IL-10 (c), IL-1*β* (d), TNF-*α* receptor (e), IL-6 receptor (f), and IL-10 receptor (g) of rats fed with control (*n* = 6–9), soy (*n* = 7–8), or fish diet (*n* = 6–10). *P < 0.05 versus control; ^#^ *P* < 0.05 soy versus fish.

The fish oil group had reduced hypothalamic protein levels of TNF-*α* (−61%) and IL-6 (−49%) when compared to the control group (Figures 2(a)-2(b)). Levels of IL-10, IL1-*β*, TNF-*α*R, and IL-6R were similar among the studied groups (Figures 2(c)–2(f)). IL-10R levels were 21% higher in the fish than in the control group (Figure 2(g)).

**Figure 3:**
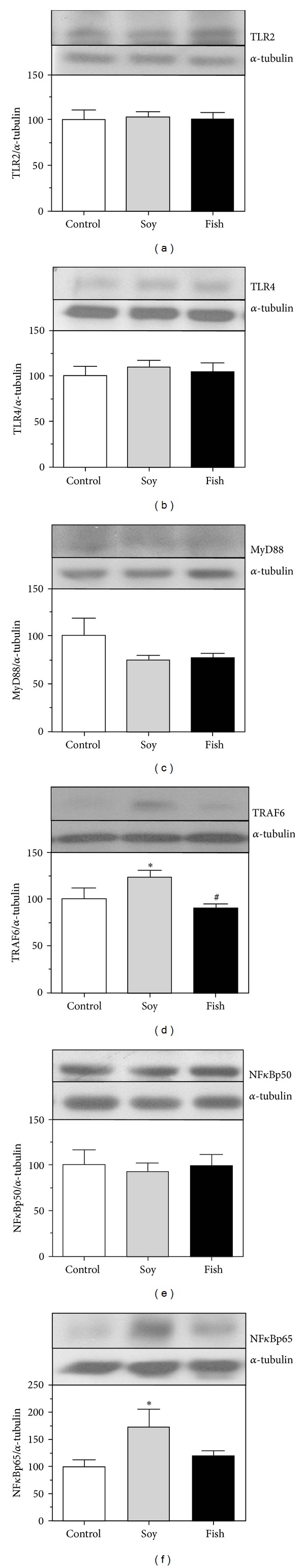
Hypothalamic protein levels of TLR2, TLR4, MyD88, TRAF-6, NF*κ*Bp50, and NF*κ*Bp65 of rats fed with control (*n* = 5–8), soy (*n* = 5–8), or fish diet (*n* = 5–8). *P < 0.05 versus control; ^#^ *P* < 0.05 soy versus fish.

No significant differences were observed among the groups in hypothalamic protein levels of TLR2, TLR4, MyD88, and NF*κ*Bp50 (Figures 3(a)–3(c) and 3(e)). TRAF6 levels were higher in the soy than those in the control and fish oil groups (25% and 27%, resp., Figure 3(d)) while NF*κ*Bp65 levels were 73% higher in the soy than those in the control group (Figure 3(f)). 

### 3.3. Inflammatory Cytokines in Liver and Gastrocnemius Muscle

**Figure 4:**
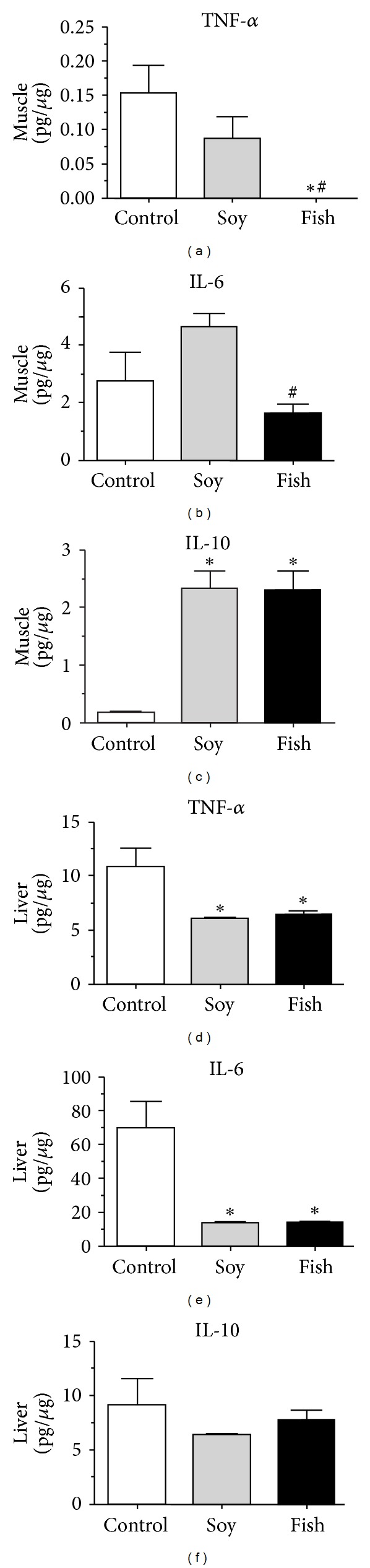
Liver and muscle protein levels of TNF-*α*, IL-6, and IL-10 of rats fed with control (*n* = 5), soy (*n* = 4), or fish diet (*n* = 4). *P < 0.05 versus control; ^#^ *P* < 0.05 soy versus fish.

Muscles TNF-*α* and IL-6 were significantly lower in the fish oil than those in the control group (Figures 4(a)-4(b)) while muscle IL-10 levels were increased in both the soy and the fish groups (Figure 4(c)). Liver levels of TNF-*α* and IL-6 were significantly lower in both the fish and the soy groups than those in the control group (Figures 4(d)-4(e)) while liver IL-10 levels were similar among groups (Figure 4(f)).

## 4. Discussion

 Since hypothalamic inflammation induced by intake of saturated and trans fat diets has been associated with hypothalamic leptin and insulin resistance and obesity, the main objective of the present study was to ascertain the effect of high-fat PUFA diets on inflammatory parameters in the hypothalamus of rats. We compared diets prepared with soy and fish oils, used to promote diet enrichment with either n-6 or n-3 series PUFAs, as previously determined [16–18].

We observed differential effects of the PUFA-rich diets. The soy diet induced increased hypothalamic levels of TRAF6 and NF*κ*Bp65, findings indicative of local stimulation of the NF*κ*B pathway. On the other hand, the fish diet led to diminished hypothalamic levels of TRAF6 and of the inflammatory cytokines TNF-*α* and IL-6, along with enhanced levels of the receptor protein for IL-10, an anti-inflammatory cytokine. These findings agree with the report of induction of inflammatory process after intracerebroventricular administration of the n-6 PUFA linoleic acid but not of the n-3 PUFA linolenic acid to rats for 3 days [6]. Interestingly, unlike the effects of saturated fat-feeding [6–9], in the present experiments, the levels of TNF-*α*, IL-1*β*, and IL-6, as well as of their receptor proteins, were not altered in the hypothalamus of the soy-diet rats, in agreement with previous data [6]. Intermediates of the NF*κ*B pathway have been shown to affect insulin signal transmission, as IKK was able to both induce serine phosphorylation of IRS-1 and upregulate SOCS3 (suppressor of cytokine signaling) in the hypothalamus of mice fed high-fat saturated diet [19]. Moreover, the inclusion of an n-3-rich oil in a saturated high-fat diet reversed the diet-induced hypothalamic inflammation and improved insulin and leptin signaling, through binding to the transmembrane G-protein-coupled receptor 120 (GPR120) [20, 21]. Since hypothalamic inflammation has been associated with central insulin resistance [6–10], the present data are also in line with our previous work, reporting impairment of hypothalamic insulin signaling and abolition of insulin hypophagia by the soy diet while the fish diet exerted a protective effect [18]. 

The present results thus showed that a PUFA-rich hyperlipidic diet based on soy oil induced an inflammatory process in the hypothalamic tissue, in a fashion similar to previously reported for saturated diets. This inflammatory status probably contributed to the impairment of feeding-controlling mechanisms and body adiposity previously reported as well as the increased body mass herein observed. On the other hand, the hypothalamus was spared of the inflammation induced by the soy diet when the excess fat was substituted for fish oil, in agreement with our earlier findings of normal insulin-induced hypophagia.

Differently from the hypothalamic tissue, in liver and muscle both the soy and the fish diets led to reduced levels of proinflammatory proteins. This agrees with data in human skeletal muscle, in which modulation of inflammatory mediators by LA (18 : 2n-6) and DHA acid (22 : 6n-3) has been associated with increased muscle fat oxidation and glucose uptake [22]. Other authors have also shown that n-3 PUFAs reduced several proinflammatory markers in human heart muscle [23, 24]. In liver, omega-3 fatty acids have been shown to reduce inflammatory mediators to prevent hepatic insulin resistance [25].

## 5. Conclusions

The results showed that the high-fat soy diet induced activation of the hypothalamic NF*κ*B inflammatory pathway, probably predisposing to the disturbances leading to obesity. On the other hand, the high-fat fish diet improved the central and the peripheral inflammatory profile via reduction of intracellular inflammatory mediators, suggesting a protection against obesity.
